# The effect of alkaline phosphatase and intrahepatic metastases in large hepatocellular carcinoma

**DOI:** 10.1186/1477-7819-11-40

**Published:** 2013-02-21

**Authors:** Jong Man Kim, Choon Hyuck David Kwon, Jae-Won Joh, Jae Berm Park, Justin Sangwook Ko, Joon Hyeok Lee, Sung Joo Kim, Cheol-Keun Park

**Affiliations:** 1 Department of Surgery, Samsung Medical Center, Sungkyunkwan University School of Medicine, #50 Ilwon-Dong Gangnam-Gu, 135-710, Seoul, Korea; 2 Department of Anesthesiology and Pain Medicine, Samsung Medical Center, Sungkyunkwan University School of Medicine, Seoul, Korea; 3 Division of Gastroenterology, Department of Medicine, Samsung Medical Center, Sungkyunkwan University School of Medicine, Seoul, Korea; 4 Department of Pathology, Samsung Medical Center, Sungkyunkwan University School of Medicine, Seoul, Korea

**Keywords:** Hepatocellular carcinoma, Liver resection, Metastasis, Survival

## Abstract

**Background:**

Hepatectomy is the standard treatment for HCC. However, large HCC poses a difficult challenge because of the technical complexity of surgical resection and the fear of postoperative hepatic decompensation. We analyzed the outcome and prognostic factors in patients with large hepatocellular carcinoma (HCC ≥10 cm) after surgery.

**Methods:**

We retrospectively investigated the medical records of 91 patients who had undergone hepatectomy between January 2006 and June 2010. A survival analysis was performed utilizing the Kaplan-Meier method and prognostic factors were evaluated using Cox regression analysis.

**Results:**

Of the 91 patients evaluated, most tumors were associated with hepatitis B virus (HBV). The median tumor size was 12.3 cm (range, 10 to 21 cm), with microvascular invasion present in most patients. The postoperative mortality rate was 2.2%. The median disease-free survival and overall survival were six months and 41 months. The one-year, two-year, and three-year disease-free survival rates were 33.5%, 29.3%, and 18.8%, respectively. The one-year, two-year, and three-year overall survival rates were 73.9%, 63.7%, and 54.8%, respectively. Of the 89 surviving patients, 69 patients (77.5%) developed HCC recurrence during the mean follow-up period of 23.4 ± 15.9 months. On multivariate analysis, the statistically significant factors that predicted HCC recurrence were ALP ≥ 80 IU/mL (*P* = 0.009) and intrahepatic metastases (*P* = 0.013).

**Conclusions:**

Our study suggests that preoperative ALP levels (≥ 80 IU/L) and intrahepatic metastases could be utilized to monitor and predict recurrence in HCC patients.

## Background

Hepatectomy has been widely accepted as the main strategy for the treatment of resectable hepatocellular carcinoma (HCC) based upon the proven impact of adequate tumor removal on prognosis. However, there continues to be debate regarding the extent of necessary resection, as well as the balance between the risks of inadequate parenchymal preservation and the benefits of oncologic clearance [1].

Despite recent advances in imaging modalities and the application of a screening program in high risk populations, such as in areas where hepatitis B is endemic, large HCC (≥ 10 cm in diameter) is still frequently encountered in clinical practice [2].

Large HCC poses a difficult challenge because of the technical complexity of surgical resection and the fear of postoperative hepatic decompensation, especially when associated with advanced cirrhosis. Treatment options for large HCC are limited. Liver transplantation is not an accepted modality for the treatment of large HCC due to issues of organ allocation and the high rates of tumor recurrence [3]. Transarterial chemoembolization (TACE) is an attractive option for large HCC, but the response rate has generally been poor and the long-term outcomes are not well known [4]. At present, provided the patient’s hepatic functional reserve is acceptable for resection, hepatectomy is considered the best option in patients with HCC ≥ 10 cm because it is potentially curative and can be performed safely with acceptable morbidity [5-7].

However, tumor recurrence is common after resection of large HCC [8,9], possibly due to unrecognized small vessel tumor invasion [5], and may signify a worse outcome as a result of currently unidentified genetic factors [10]. Overall patient survival after hepatectomy usually depends upon the outcome following the liver resection, as well as additional treatments for recurrence.

We retrospectively analyzed the long-term outcomes of patients with large HCC following hepatectomy at a single center and evaluated the prognostic factors that influenced tumor recurrence in these patients.

## Methods

### Patients

From January 2006 to June 2010, 91 patients with large HCC (≥ 10 cm in diameter) underwent hepatectomy at Samsung Medical Center. Younger patients (< 18 years of age), pathologically-proven mixed hepatocellular carcinoma and cholangiocarcinoma, and patients who were lost to follow-up after the hepatectomy were excluded from this study. The demographics, preoperative laboratory results, and pathologic data of all of the patients were collected from the electronic medical records (EMR) and retrospectively reviewed. Liver function was evaluated using the Child-Pugh classification system.

### Surgery and pathology

Preoperative evaluation of liver function included serum levels of bilirubin, transaminases, alkaline phosphatase, albumin, and prothrombin time. Selection criteria for the hepatectomy depended on the extent and location of the tumor, liver function, indocyanine green retention (ICG) test results, and the volume of the future liver remnant. Child-Pugh class C, severe comorbidity, and distant metastases were considered contraindications for hepatectomy.

A standard operative technique for hepatectomy was utilized for these tumors. Depending on the part of the liver to be resected, adequate mobilization was performed. An anterior approach was used in patients for whom right lobe mobilization was considered hazardous [11]. Selective clamping of the portal vein and hepatic artery was performed when feasible. If this was not possible, an intermittent Pringle maneuver was performed instead. Parenchymal transection was performed using CUSA (Cavitron Ultrasonic Surgical Aspirator) under low central venous pressure.

Postoperative histological assessment and reporting included the maximal tumor diameter, capsular formation, capsular invasion, portal vein invasion, bile duct invasion, microvascular invasion, serosa involvement, intrahepatic metastasis, multicentric occurrence of HCC and others. Intrahepatic metastasis and multicentric occurrence were defined based on guidelines from the Liver Cancer Study Group of Japan [12]. Histologic grade of HCC was assessed according to the Edmonson-Steiner grading system [13], and grouped as well-differentiated (grade I), moderately-differentiated (grade II), or poorly-differentiated (grades III and IV).

### Surveillance after surgical resection

Postoperative mortality was defined as all deaths within 30 days of hepatectomy. After surgery, patients were followed postoperatively every two to three months. Follow-up parameters included physical examination, serum alpha-fetoprotein (AFP), protein induced by vitamin K antagonist II (PIVKA-II), liver function tests, and chest x-rays. Abdominal computed tomography (CT) was performed every three months or when recurrence was suspected. Magnetic resonance imaging (MRI) and/or positron emission tomography (PET) scans were performed if CT did not show definitive evidence of recurrence. Detailed information on patients found to have a recurrence was recorded. Patients with intrahepatic recurrences were treated with radiofrequency ablation (RFA), TACE, or sorafenib according to their functional liver reserve and the pattern of recurrence. The follow-up time was considered to be the length of time from the surgery to the last follow-up (1 December 2011) or death. No patients were lost to follow-up and all 91 patients were included in the survival analysis.

### Statistical analysis

All data were analyzed using SPSS statistical software (Version 19.0; SPSS Inc., Chicago, IL, USA). Continuous variables were presented as median and range and compared by using the Mann–Whitney *U* test. Categorical variables were compared using Fisher’s exact test. The arbitrary cut-off value in each continuous variable was determined by the receiver operating characteristics (ROC) curve. The disease-free survival rates and overall survival rates were calculated with the Kaplan-Meier method and compared using the log-rank test. Univariate analyses were performed to identify risk factors of HCC recurrence in large HCC using a Cox regression model. A backward multivariate analysis was performed using a Cox proportional hazard model on all variables that were significantly associated with survival on univariate analysis. A *P*-value < 0.05 was considered statistically significant.

## Results

### Patient demographics and tumor characteristics

Clinicopathological features of the 91 patients are summarized in Table 1. All patients were Child-Pugh class A and none received preoperative radiation. The majority of the patients were male (87.9%) and the median age was 52 years (range, 19 to 82). The etiology of most of the HCC cases was hepatitis B virus (HBV). The median AFP and PIVKA-II were 134.4 ng/mL (range, 1.8 to 200,000 ng/mL) and 500 mAU/mL (range, 5 to 2000 mAU/mL), respectively. Eighteen patients (19.8%) were treated with TACE prior to hepatic resection.

**Table 1:** Clinicopathologic features and surgical treatments of patients with large hepatocellular carcinoma

**Gender**	
Male	80 (87.9%)
Female	11 (12.1%)
Age (years)	52 (19 to 82)
Etiology	
HBV	60 (65.9%)
HCV	2 (2.2%)
Alcohol	4 (4.4%)
non-B, non-C	21 (23.1%)
Others	3 (3.3%)
AFP (ng/dL)	
< 1000	58 (63.7%)
≥ 1000	33 (36.3%)
PIVKA-II (mAU/mL)	
< 200	28 (30.8%)
≥ 200	63 (69.2%)
White blood cells (/uL)	6,040 (2,100 to 10,790)
Hemoglobin (g/dL)	14.2 (8.1 to 17.0)
Platelet counts (/uL)	205,000 (61,000 to 627,000)
INR	1.07 (0.91 to 1.31)
Albumin (g/dL)	4.0 (2.9 to 5.0)
Total bilirubin (mg/dL)	0.7 (0.3 to 1.2)
AST (U/L)	43 (14 to 353)
ALT (U/L)	39 (12 to 385)
ALP (U/L)	92 (38 to 287)
Creatinine (mg/dL)	0.89 (0.65 to 2.99)
Maximum tumor size (cm)	12.3 (10 to 21)
Cirrhosis	17 (18.7%)
Grade	
1 and 2	75 (82.4%)
3 and 4	16 (17.6%)
Capsular invasion	77 (84.6%)
Microvascular invasion	85 (93.4%)
Portal vein invasion	19 (20.9%)
Bile duct invasion	4 (4.4%)
Serosa involvement	8 (8.8%)
Intrahepatic metastasis	30 (33.0%)
Multicentric occurrence	5 (5.5%)

*HBV*, hepatitis B virus; *HCV*, hepatitis C virus; *AFP*, alpha-fetoprotein; *PIVKA-II*, protein induced by vitamin K antagonist-II; *TACE*, transarterial chemoembolization; *INR*, international normalized ratio; *AST*, aspartate aminotransferase; *ALT*, alanine aminotransferase; *ALP*, alkaline phosphatase.

Perioperative and postoperative characteristics were outlined in Table 2. The median blood loss during operation and operation time were 550 mL (range, 100 to 4000 mL) and 330 minutes (150 to 720 minutes), respectively.

**Table 2:** Perioperative and postoperative characteristics

**Perioperative**	
Type of operation	
Anatomical	80 (87.9%)
Non-anatomical	11 (12.1%)
Hepatectomy	
Right hepatectomy	55 (60.4%)
Left hepatectomy	1 (1.1%)
Posterior segmentectomy	2 (2.2%)
Anterior segmentectomy	1 (1.1%)
Extended right hepatectomy	11 (12.1%)
Extended left hepatectomy	3 (3.3%)
Left lateral segmentectomy	5 (5.5%)
Central hepatectomy	2 (2.2%)
Segmentectomy	11 (12.1%)
Blood loss during operation (mL)	550 (100 to 4000)
Operation time (minutes)	330 (150 to 720)
**Postoperative**	
Morbidity	
Wound infection	6 (6.6%)
Atelectasis	8 (8.8%)
Pneumonia	2 (2.2%)
Pleural effusion	4 (4.4%)
Bile leakage	6 (6.6%)
Ascites	8 (8.8)
Delirium	1 (1.1%)
Hepatic failure	1 (1.1%)
Hyperbilirubinemia	1 (1.1%)
Mechanical obstruction	1 (1.1%)
Portal vein thrombosis	1 (1.1%)
Spontaneous bacterial peritonitis	1 (1.1%)
Wound dehiscence	2 (2.2%)
Mortality	2 (2.2%)

The median tumor size was 12.3 cm (range, 10 to 21 cm). Portal vein invasion and bile duct invasion were detected in 19 (20.9%) and 4 (4.4%) patients, respectively. Microvascular invasion was seen in 85 patients (93.4%) and capsular invasion was seen in 77 patients (84.6%). Intrahepatic metastasis and multicentric occurrence were detected in 30 (33.0%) and 5 patients (5.5%), respectively.

Eight-nine of the 91 patients recovered from hepatectomy, while two died from liver failure after the surgery. The median length of hospitalization after hepatectomy was ten days (range, seven to fifty-three days) and the mean duration of follow-up was 23.4 ± 15.9 months.

### Survival

**Figure 1:**
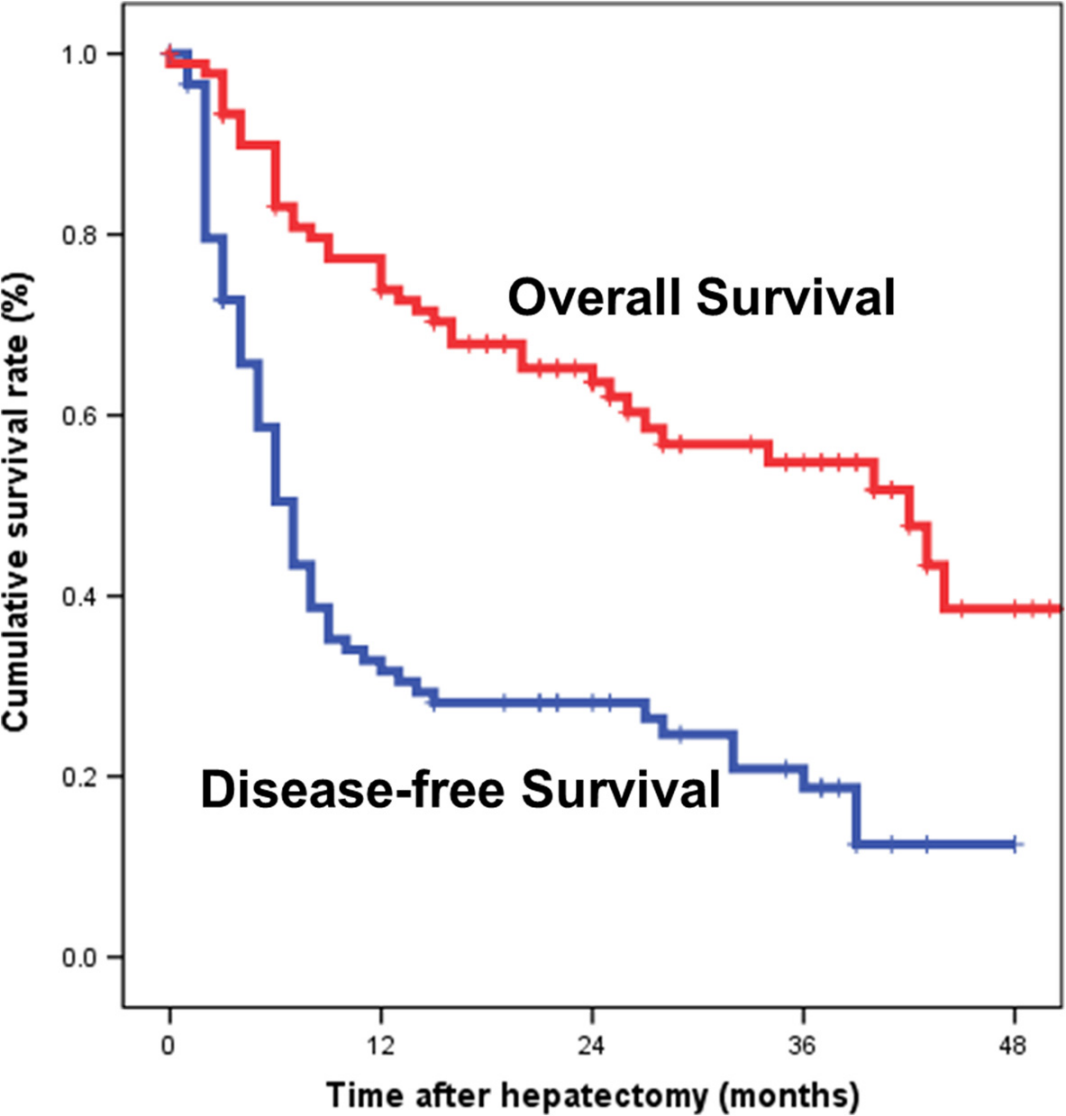
Disease-free survival and overall survival curves for patients who underwent hepatic resection of hepatocellular carcinoma larger than 10 cm in diameter.

The median disease-free survival and overall survival of the patients who underwent resection for large HCC were six and 41 months, respectively. The one-year, two-year, and three-year disease-free survival rates were 33.5%, 29.3%, and 18.8%, respectively. The one-year, two-year, and three-year overall survival rates were 73.9%, 63.7%, and 54.8%, respectively (Figure 1).

### Recurrence of hepatocellular carcinoma

Of the 89 surviving patients, 69 patients (77.5%) developed a HCC recurrence during a mean follow-up of 23.4 ± 15.9 months. Twenty-one of the 69 (30.4%) presented initially with an isolated intrahepatic recurrence, 14 patients (20.3%) presented with an extrahepatic recurrence, and 34 patients (49.3%) presented with concurrent intrahepatic and extrahepatic recurrences. The lung (n = 29, 42.0%) and liver (n = 27, 39.1%) were the main recurrence sites. Sixty-three (91.3%) of the 69 patients received treatments, such as TACE, RFA, and sorafenib, after tumor recurrence. No patients received selective internal radiation therapy after recurrence.

### Prognostic factors

**Figure 2:**
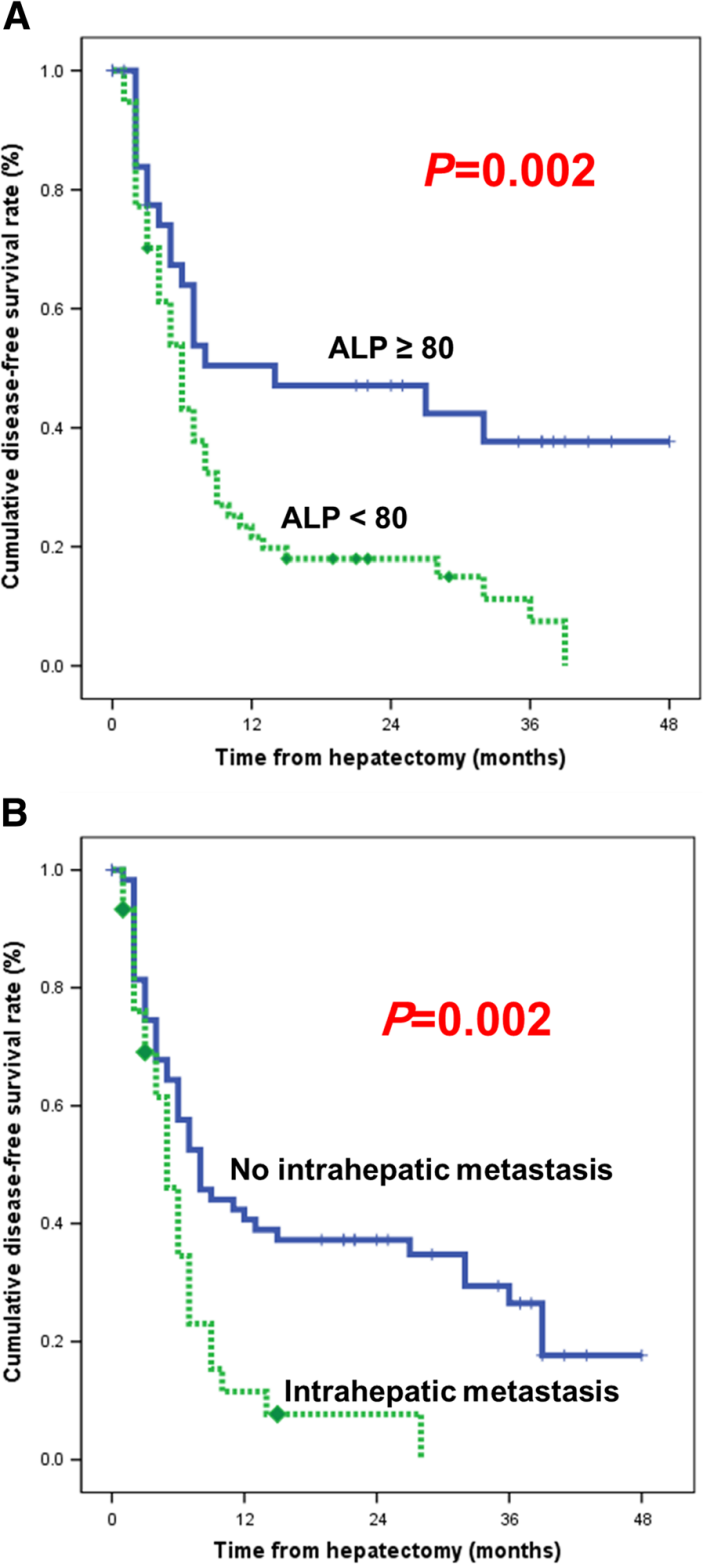
(A) Influence of ALP levels and (B) intrahepatic metastasis on disease-free survival.

Univariate analysis of prognostic factors for disease-free survival following hepatic resection in patients with large HCC identified AFP ≥ 1000 ng/mL, decreased serum albumin levels, increased ALP (normal range, 53 to 128 IU/mL) levels, and intrahepatic metastases as statistically significant factors determining poor prognosis (Table 3). Gender, age, serum PIVKA-II, type of operation (non-anatomical), tumor size, tumor grade, capsular invasion, microvascular invasion, portal vein invasion, bile duct invasion, serosa involvement, and multicenteric occurrence were not statistically significant. On multivariate analysis, the statistically significant factors predicting HCC recurrence were ALP ≥ 80 IU/mL (odds ratio (OR), 2.075; 95% confidence interval (CI), 1.197 to 3.599; *P* = 0.009) and intrahepatic metastases (OR, 1.924; 95% CI, 1.149 to 3.221; *P* = 0.013). The impact of ALP and intrahepatic metastases are depicted in Figure 2.

**Table 3:** Univariate analysis of risk factors related to tumor recurrence in patients with large HCC after hepatic resection

**Variables**	**Odds ratio**	**95% confidence interval**	***P*-value**
Gender - Female	1.076	0.531 to 2.182	0.838
Age	0.999	0.980 to1.019	0.933
AFP ≥ 1000	1.935	1.179 to 3.176	0.009
PIVKA-II ≥ 200	1.136	0.677 to 1.906	0.630
White blood cells	0.933	0.813 to 1.071	0.325
Hemoglobin	0.884	0.782 to 1.001	0.051
Platelet count	1.002	1.000 to 1.004	0.075
INR	2.822	0.154 to 51.533	0.484
Albumin	0.443	0.231 to 0.850	0.014
Total bilirubin	1.162	0.570 to 2.367	0.680
AST	1.002	0.998 to 1.006	0.313
ALT	1.000	0.996 to 1.004	0.919
ALP > 80 IU/mL	2.216	1.282 to 3.830	0.004
Glucose	0.998	0.993 to 1.004	0.998
Creatinine	0.787	0.368 to 1.683	0.537
Type of operation (non-anatomical)	1.402	0.711 to 2.768	0.330
Maximum tumor size	0.990	0.990 to 1.008	0.780
Cirrhosis	1.690	0.926 to 3.084	0.087
Grade (3 and 4)	1.141	0.612 to 2.130	0.678
Capsular invasion	0.532	0.277 to 1.020	0.057
Microvascular invasion	1.812	0.566 to 5.797	0.317
Portal vein invasion	1.642	0.933 to 2.889	0.085
Bile duct invasion	1.184	0.371 to 3.785	0.775
Serosa involvement	1.142	0.457 to 2.851	0.776
Intrahepatic metastasis	2.019	1.265 to 3.517	0.004
Multicentric occurrence	0.481	0.118 to 1.966	0.308

*AFP*, alpha-fetoprotein; *PIVKA-II*, protein induced by vitamin K antagonist-II; *TACE*, transarterial chemoembolization; *INR*, international normalized ratio; *AST*, aspartate aminotransferase; *ALT*, alanine aminotransferase; *ALP*, alkaline phosphatase.

### ALP levels and intrahepatic metastases in patients

**Figure 3:**
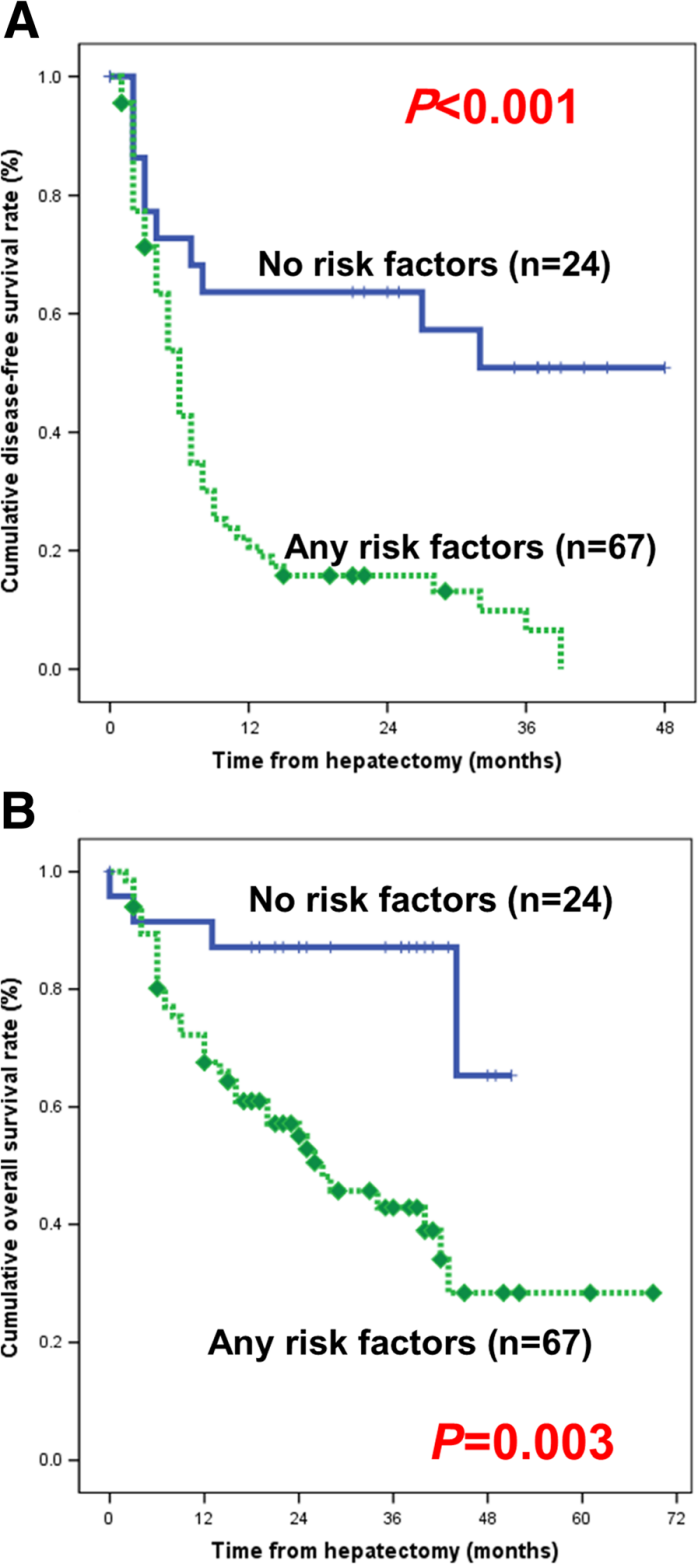
(A) Disease free survival and (B) overall survival in patients with no risk factors when compared with those who had at least one risk factor.

We compared the 24 patients who had neither of the two risk factors (ALP ≥ 80 IU/L or intrahepatic metastases) with the 67 patients who had at least one risk factor. Those with no risk factors had a one-year disease-free survival rate of 63.6% and a one-year overall survival rate of 91.5%. In contrast, the patients with at least one risk factor had a one-year disease-free survival rate of 20.6% and a one-year overall survival rate of 72.3% (Figure 3). The differences in the disease-free survival and overall survival rates were statistically significant (*P* < 0.001 and *P* = 0.003, respectively).

## Discussion

During the past decade, hepatectomy for large HCC has evolved into a safe procedure with a low operative mortality [7,14]. The 30-day mortality rate of 2.2% (n = 2) in this study is lower than that reported in general, which is roughly 5 to 10% [15]. Among postoperative mortality cases, one case had undergone left extended hepatectomy with the cause of death being right hepatic artery dissection during Pringle’s maneuver. Although the right hepatic artery had been anastomosed with the right gastroepiploic artery, liver failure developed. The other case had undergone right hepatectomy and although the preoperative ICG test was 16.7%, liver failure developed after the hepatectomy.

However, the long-term survival remains unsatisfactory predominately because of the high incidence of recurrence and metastases after hepatectomy [16]. Our study also revealed a high incidence of recurrence with one-year, two-year, and three-year disease-free survival rates of 33.5%, 29.3%, and 18.8%, respectively.

The greater sizes of these larger HCCs indicate that the lesion is already advanced, with a greater possibility of tumor spread, including the existence of satellite nodules or macrovascular invasion [5]. Such advanced tumors carry a higher risk of recurrence even after hepatectomy, such that the benefit of hepatic resection becomes marginal [17].

With the improvements in surgical techniques and perioperative care, there has been a significant improvement in the postoperative outcome for patients following liver resection in large volume centers [2,18]. This has encouraged surgeons to consider surgical resection for large tumors. We followed an aggressive policy of surgical resection in the patients with large HCC as we felt that it provided significant local control with a good quality of life and the potential for cure in an otherwise hopeless situation. In the present study, the three-year overall survival rate was 54.8%, indicating an improved outcome when compared with rates of between 16.7% and 33% in recent reports [1,2,7,14,17-19].

The size of HCC has traditionally been considered an important risk factor for patient survival. However, the size of a single HCC mass in the absence of vascular invasion is no longer regarded as a critical factor [20]. Furthermore, in 2005, the American Association for the Study of Liver Disease suggested that the size of a tumor alone is not a limiting factor for surgical resection [21]. A non-cirrhotic liver can tolerate resection of up to 80% of its volume. The regenerative capacity of the liver enables functional compensation within a few weeks with regeneration of approximately 75% of the preoperative liver volume within one year [22,23].

Mok *et al*. reported that extrahepatic recurrence occurred in 43.4% patients [17], while Poon *et al*. reported that extrahepatic recurrence was significantly more frequent in patients with hepatic resection for HCC ≥ 10 cm (31.9%) than those with HCC < 10 cm (12.8%) [6]. Recently, Yamashita *et al*. reported that extrahepatic recurrence, such as lung, bone, brain, and peritoneum, was significantly more frequent in patients with hepatic resection for HCC ≥ 10 cm than those for HCC < 10 cm [24]. The present study reported that about two-thirds of initial HCC recurrences were extrahepatic (with or without intrahepatic recurrence), while the other one-third were isolated intrahepatic recurrences.

TACE has been used after surgery to treat intrahepatic HCC recurrence, particularly in patients with a multifocal recurrence within the remaining liver [25]. Percutaneous RFA is another safe and effective procedure for treating intrahepatic recurrences [26]. Repeat liver resection is indicated for patients with well-preserved liver function [6,25]. In our study, 8.7% (n = 6) of patients with recurrent HCC were not treated for tumor recurrence because of poor general condition or rapid tumor spread to multiple organs.

Increased preoperative serum ALP levels and intrahepatic metastasis were predisposing factors for tumor recurrence after hepatectomy in patients with large HCC. The tumor size, differentiation of the tumor, and the margin of resection were not significant predictors of tumor recurrence. The small number of patients with a positive margin (n = 5) in our study makes it difficult to draw a firm conclusion regarding the importance of margins.

Intrahepatic metastasis is thought to be the main mechanism of early recurrence based on histological analysis of tumor recurrence [27]. ALT is a well-known marker of inflammatory necrosis in the liver [28,29]. Persistent inflammation will not only cause necrosis and regeneration of hepatocytes, thereby leading to DNA instability in the hepatocytes and causing the HCC to occur more frequently, but will also enhance the development of intrahepatic metastasis by up-regulating the expression of vascular adhesion molecules [30]. Therefore, suppression of ALT elevation by treatment with anti-inflammatory drugs has been proven to delay recurrence after hepatectomy, thereby providing an important strategy for the prevention of early recurrence [31,32]. Hepatocarcinogenesis by HBV is associated with ALP as an inflammatory marker. Cumulative data derived from Asian populations with HCC revealed that elevation of the ALP level was associated with poor outcomes [33,34]. A large-scale study in Taiwan demonstrated that ALP could predict the outcome, while a Western study of Asian Americans with HCC also reported that AFP and ALP were independent predictors of survival [33,34].

## Conclusions

In conclusion, hepatectomy for large HCC is safe in carefully selected patients. However, we expect a greater likelihood of early tumor recurrences in patients with large HCC who have high preoperative ALP levels (≥ 80 IU/L) and intrahepatic metastases after hepatectomy. Our study suggests that preoperative ALP levels and intrahepatic metastases could be utilized to monitor and predict recurrence in HCC patients.

## Abbreviations

HCC: Hepatocellular carcinoma;EMR: Electronical medical records;ICG: Indocyanine green;CUSA: Cavitron Ultrasonic Surgical Aspirator;AFP: Alpha-fetoprotein;PIVKA-II: Protein induced by vitamin K antagonist II;CT: Computed tomography;MRI: Magnetic resonance imaging;PET: Positron emission tomography;RFA: Radiofrequency ablation;TACE: Transarterial chemoembolization;ROC: Receiver operating characteristics;ALP: Alkaline phosphotase

## Competing interest

The authors do not have any conflicts of interest or financial disclosures to report.

## Authors’ contributions

JMK was responsible for design, acquisition of data, analysis data, interpretation of data, and writing. CHDK and J-WJ were responsible for design and interpretation of data. JBP and JSK were responsible for acquisition of data and interpretation of data. JHL, SJK, and C-KP were responsible for analysis and interpretation of data. All authors read and approved the final manuscript.
